# Supplementary material for: Enhanced Visible-Light-Driven Photocatalysis of Ag/Ag2O/ZnO Nanocomposite Heterostructures
Source: Nanomaterials (Basel). 2022 Jul 23;12(15):2528. doi: 10.3390/nano12152528 (PMC9330815; doi:10.3390/nano12152528)
Supplement: Supplementary file 1 [file nanomaterials-12-02528-s001.zip › nanomaterials-1834996-supplementary.pdf]

nanomaterials

Supporting information:

Enhanced Visible-Light-Driven Photocatalysis of $Ag/Ag_2O/ZnO$ Nanocomposite Heterostructures

Chadrasekhar Loka * and Kee-Sun Lee *

Department of Advanced Materials Engineering & Smart Natural Space Research Center, Kongju National University, Cheonan 31080, Korea
* Correspondence: csloka89@gmail.com (C.L.); kslee@kongju.ac.kr (K.-S.L.)

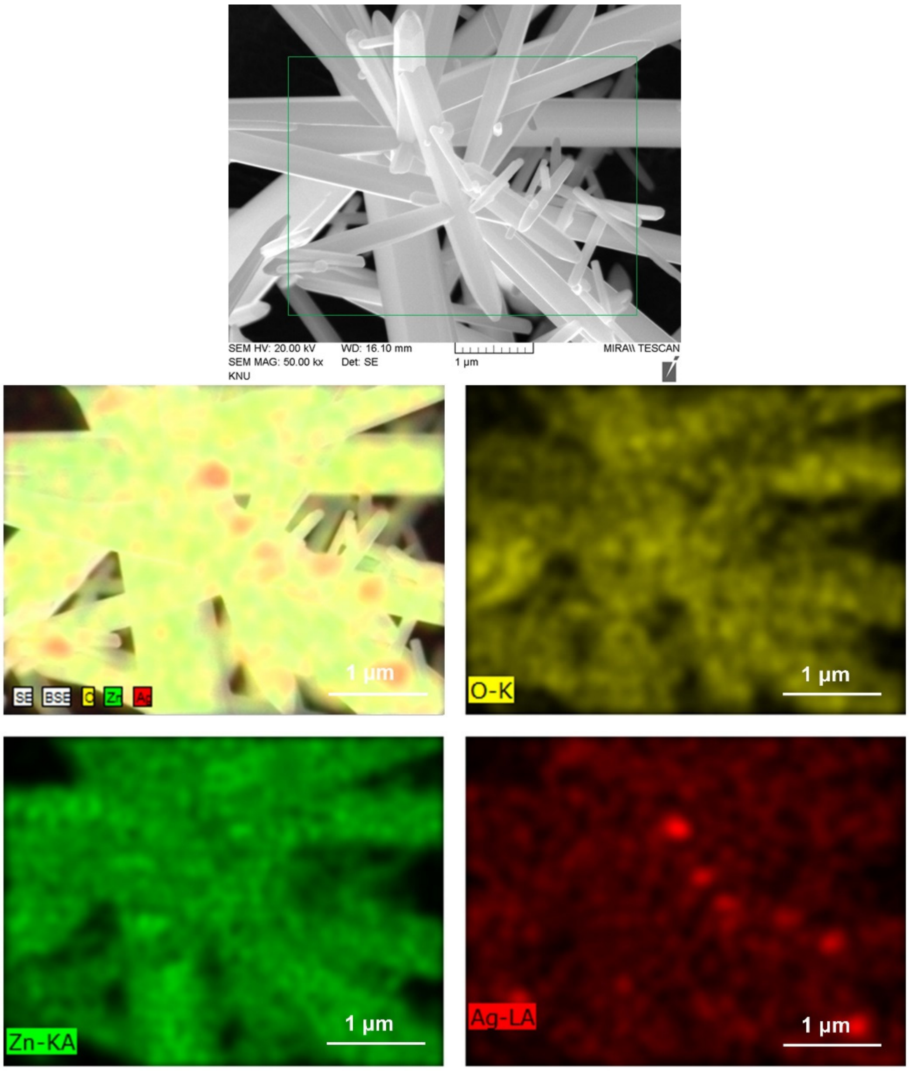

Figure S1. SEM image with corresponding EDX elemental mapping images of Zn, Ag, and O correspond to the 5% Ag-ZnO photocatalyst.

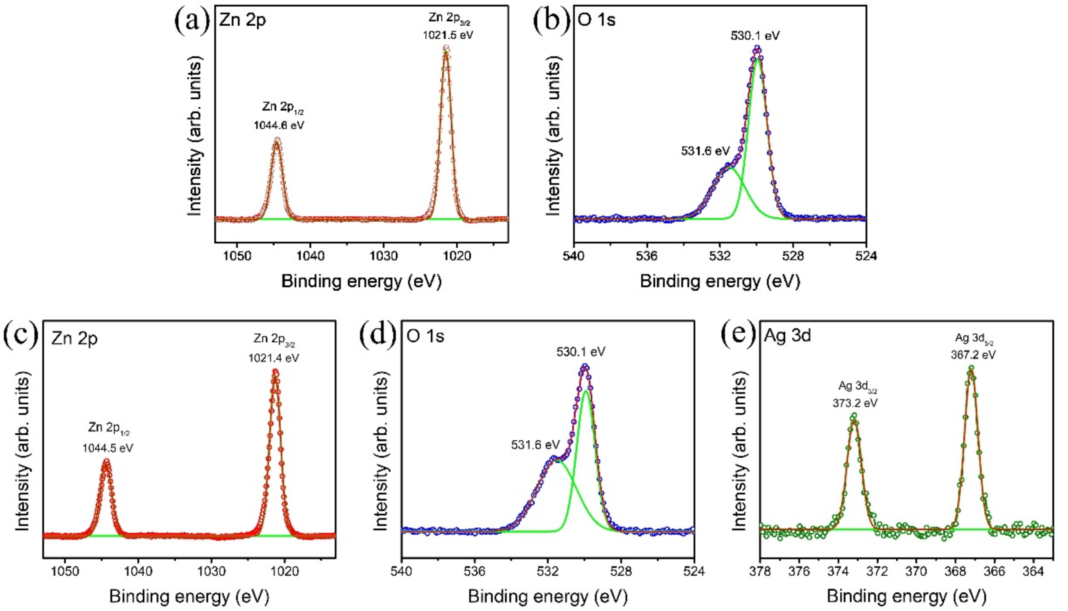

Figure S2. High-resolution XPS spectra of the pure ZnO (a, b), and 2% Ag-ZnO (c-e).

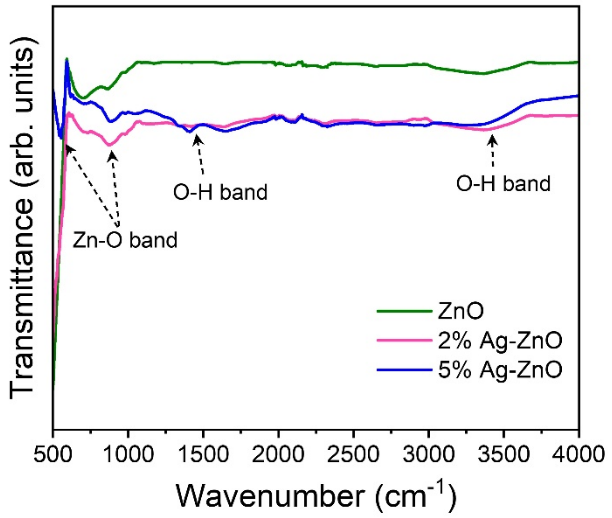

Figure S3. FTIR spectra of the pure ZnO, 2% Ag-ZnO, and 5% Ag-ZnO samples.

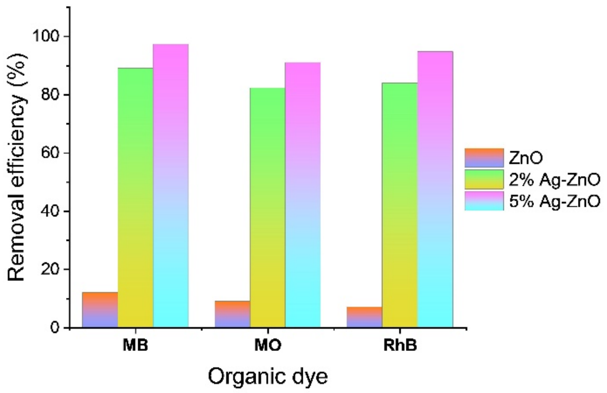

Figure S4. Photocatalytic removal efficiency of the ZnO, 2% Ag-ZnO, and 5% Ag-ZnO within 60 min under visible light irradiation for methylene blue (MB), methyl orange (MO), and rhodamine (RhB).

Table S1. Photocatalytic performance of the pure ZnO, 2% Ag-ZnO, and 5% Ag-ZnO under visible light irradiation for different organic dyes MB, MO, and RhB.

Sample	Dye degradation efficiency (%)			Rate constant, k (min^{-1})		
	**MB**	**MO**	**RhB**	**MB**	**MO**	**RhB**
ZnO	12	9	7	0.2×10^{-2}	0.1×10^{-2}	0.09×10^{-2}
2%Ag-ZnO	89.1	82.3	84.0	3.6×10^{-2}	2.8×10^{-2}	3.1×10^{-2}
5%Ag-ZnO	97.3	91.1	94.8	5.7×10^{-2}	3.7×10^{-2}	5.1×10^{-2}
